# Neural Correlates of Unsuccessful Memory Performance in MCI

**DOI:** 10.3389/fnagi.2014.00201

**Published:** 2014-08-13

**Authors:** N. Chechko, E. I. Drexler, B. Voss, T. Kellermann, A. Finkelmeyer, F. Schneider, U. Habel

**Affiliations:** ^1^Department of Psychiatry, Psychotherapy and Psychosomatics, Medical School, RWTH Aachen University, Aachen, Germany; ^2^Jülich Aachen Research Alliance (JARA) – Translational Brain Medicine, Jülich and Aachen, Germany; ^3^Institute of Neuroscience, Newcastle University, Campus for Ageing and Vitality, Newcastle upon Tyne, UK

**Keywords:** Alzheimer’s disease, attention, fMRI, memory, mild cognitive impairment, unsuccessful encoding

## Abstract

People with mild cognitive impairment (MCI) are at an elevated risk of developing Alzheimer’s disease or other forms of dementia. Although the neural correlates of successful memory performance in MCI have been widely investigated, the neural mechanisms involved in unsuccessful memory performance remain unknown. The current study examines the differences between patients suffering from stable amnestic MCI with multiple deficit syndromes and healthy elderly controls in relation to the neural correlates of both successful and unsuccessful encoding and recognition. Forty-six subjects (27 controls, 19 MCI) from the HelMA (Helmholtz Alliance for Mental Health in an Aging Society) completed a comprehensive neuropsychological test battery and participated in an fMRI experiment for associative face-name memory. In patients, the areas of frontal, parietal, and temporal cortices were less involved during unsuccessful encoding and recognition. A temporary dysfunction of the top-down control of frontal or parietal (or both) areas is likely to result in a non-selective propagation of task-related information to memory.

## Introduction

Mild cognitive impairment (MCI) is a term used to describe a condition involving problems in cognitive function (mental abilities such as thinking, knowing, and remembering) and is considered a risk factor for the development of dementia. As MCI is thought to constitute a transitional state between healthy aging and dementia, patients with MCI display greater cognitive dysfunction than expected for their individual age, albeit without fulfilling the criteria for the diagnosis of dementia (Gauthier et al., 2004; Winblad et al., 2004; Petersen and Negash, 2008). Epidemiological studies suggest that the progression of MCI is heterogeneous, and may be reversible, and that the prognosis greatly depends on the subtype of the MCI (DeCarli, 2003). Based on neuropsychological profile, MCI can be classified into four subtypes: single amnestic, multiple amnestic, single non-amnestic, and multiple non-amnestic (Busse et al., 2006). According to some recent studies (Rizzo et al., 2000; Gauthier et al., 2004; Murray and Ranganath, 2007; Dickerson and Sperling, 2008; Kim et al., 2012; Drexler et al., 2013), the most common form of MCI is a multiple deficit syndrome with memory impairment. Amnestic MCI has also been found to progress preferentially to Alzheimer’s disease (AD), with a 10–15% annual risk of conversion (Portet et al., 2006). Longitudinal investigations have shown that after 3 years, 55% of total 165 amnestic MCI patients had progressive difficulty with memory, with 19% of the cases converting to AD (Daly et al., 2000).

With the development of neuroimaging techniques, the investigation of indicative characteristics in genetic, clinical, and biological areas of research has been expanded to the neural correlates of MCI and impaired memory functions, the leading symptom of amnestic MCI. The neural correlates of memory impairment in amnestic MCI range from dysfunctions in the medial temporal lobe including the hippocampus and the amygdala to impairments in the entorhinal cortex, the occipital lobe, the precuneus, the posterior cingulate gyrus, and the prefrontal cortex (Ries et al., 2008). In an associative encoding task, patients with amnestic MCI (compared to control subjects) hyperactivated the hippocampus, whereas patients with AD showed decreased entorhinal and hippocampal activation patterns (Dickerson et al., 2005). Celone et al. (2006) corroborated these findings by showing that amnestic MCI patients with minor cognitive deficits had elevated levels of activation in the hippocampus as opposed to hypoactivation of the hippocampus seen in more impaired patients. Results from a significant number of studies (Sperling et al., 2003; Hämäläinen et al., 2007; Clément and Belleville, 2010; O’Brien et al., 2010; Kim et al., 2012) suggest that patients are capable of compensating for their deficits in the early stages of amnestic MCI, whereas in later stages the ability to recruit helpful additional resources is lost.

Recent studies have shown that the default network regions that converge on the prefrontal areas and the posterior cingulate extending into the precuneus (Greicius et al., 2009) are involved in conditions associated with memory impairment. *In vivo* amyloid and fMRI imaging suggest that elevated amyloid deposition and relatively poor memory performance are linked to aberrant default network functional activity in asymptomatic, minimally impaired older individuals (Vannini et al., 2013).

Previously, functional magnetic resonance imaging (fMRI) was tackled largely with the neural correlates of memory encoding and memory retrieval by means of block-design paradigms (Sperling et al., 2003; Dickerson et al., 2004; Bondi et al., 2005; Miller et al., 2008; Trivedi et al., 2008). More recently, fMRI studies on memory in MCI have employed event-related designs, thus accounting for a more specific understanding of successful memory functioning. For example, Heun et al. (2007) have found that during successful memory performance patients with amnestic MCI show elevated prefrontal cortex activation as compared to control subjects. Kircher et al. (2007) have found elevated hippocampal activation associated with successful encoding in amnestic MCI patients.

Given that the diagnosis of amnestic MCI is contingent on the assumption that one or more cognitive processes are impaired, investigating the potential difference between MCI and healthy controls in the context of cognitive failure is highly relevant. In this regard, the investigation of unsuccessful memory is particularly pertinent as it is not as well understood as successful memory. Stevens et al. (2008) have found that while decreased hippocampal activity accompanies unsuccessful encoding in both younger and older adults, in older adults there is increased additional activity indicating distraction from task-irrelevant input (such as scanner-noise). The aim of our study was to investigate potential differences between patients with MCI and healthy elderly controls with regard to the neural correlates of successful versus unsuccessful encoding and recognition. Only those amnestic MCI patients (with multiple deficit syndromes) who had been stable for at least 1 year were included in the study.

Based on previous results, we expected to find increased activity during successful memory encoding and recognition in patients with MCI, compared to the control subjects, in memory-related brain areas. We hypothesized that encoding and recognition failure would result in a decreased activation pattern of these brain areas in patients with MCI relative to elderly controls.

Most fMRI studies investigating MCI employ a very brief neuropsychological test battery, often using only screening tests to diagnose MCI (Celone et al., 2006; Johnson et al., 2006; Petrella et al., 2006; Heun et al., 2007; Kircher et al., 2007). Previous results, for the most part, have been based on single verification of group assignments, risking the consequence of subsequent reconversion of some MCI patients. Our work, on the other hand, as part of a multiple-assessment longitudinal project on predictors of dementia, was based on an extensive neuropsychological test battery, which assigned the study sample either to the group of patients with MCI or the group of healthy elderly controls (Drexler et al., 2013).

## Materials and Methods

### Participants

A total sample of 81 elderly participants (45 controls, 36 MCI) were examined at the first of five measurement time points of the fMRI part of the Helmholtz Alliance for Mental Health in an Aging Society (HelMA) longitudinal study to establish neurobiological predictors for the development of dementia. They were recruited through newspaper advertisement and from local facilities for the elderly. All were paid for their participation and gave written informed consent. The study was approved by the Institutional Review Board (IRB) of the Medical Faculty, RWTH Aachen University, according to the Helsinki declaration. Each subject underwent extensive neuropsychological examination prior to group assignment (MCI vs. control group). Criteria for the diagnosis of MCI were oriented on Winblad et al. (2004). Subjects were considered MCI when they had (1) an impaired score of at least 1.5 SD below the mean according to the normative data set of at least one cognitive test, (2) no dementia, and (3) preserved activities of daily living. A broad description of the neuropsychological test battery was used (Table 1). All subjects were screened for suitability of fMRI participation and right-handedness according to the Edinburgh inventory for handedness (Oldfield, 1971). None of the subjects had any history of psychiatric or neurological diseases or drug addiction. Subjects with unexplained mild tinnitus or rotatory vertigo were deemed suitable for participation, as long as they did not feel subjectively disadvantaged. Thirteen participants (six controls, seven MCI) with recognition performance less than or equal to 50% (below/equal to chance-level) as well as 13 participants (six controls, seven MCI) with unstable diagnoses (as reappraised in the second measurement time point) were excluded from analyses. An additional nine subjects were not included due to excessive head movement (seven subjects; translation >3 mm), misunderstanding of the task, or an anatomical abnormality of the frontal cortex. The final sample consisted of 27 healthy elderly controls and 19 patients with MCI. For detailed information with regard to basic subject characteristics and a selection of neuropsychological test performance, please refer to Table 1.

**Table 1:** **Subject characteristics and neuropsychological test performance**.

Subject characteristics	Controls (*n* = 27)	MCI (*n* = 19)
Age	68.7 (4.0)	71.5 (7.9)
Education	10.7 (1.8)	9.7 (2.4)
^a^MWT-B score	33 (2.2)	31.6 (4.5)
^b^BDI-score	4.0 (4.0)	3.4 (3.2)
^c^BADL-score	1.2 (0.3)	1.2 (0.3)
Male	7	7
Hypertonia	12	12

*Values are presented as means (SD)*.

*^a^Multiple-choice word test, German version*.

*^b^Beck’s Depression Inventory, German version*.

*^c^The Bayer Activities of Daily Living Scale*.

### Experimental design

All subjects participated in two separate experimental tasks, memory encoding, and memory recognition. Both tasks were presented using Presentation software package 10 (Neurobehavioral systems Inc., San Francisco, CA, USA).

#### Memory encoding

The first task intended to measure the neural correlates of memory encoding by means of learning-associated face-name pairs. Subjects were shown a total of 20 unfamiliar and emotionally neutral faces drawn from the facial expressions for brain activation (FEBA) database [16]. The faces covered all age ranges and had an equal gender ratio. All names were common, two-syllable German first names. Each face was presented four times: twice together with the to-be-learned name (associative version) and twice together with the word “name” in brackets (control version), both either below the right or the left corner of the picture. We conducted one repetition of each face/name pair given the fact that in our unpublished behavioral pilot study, the recognition performance of the majority of healthy controls had remained either below or at chance-level when the face/name pair was presented only once. These repetitions started to appear only after all 20 faces had been shown once in each of the two task versions and their order was newly randomized.

**Figure 1:**
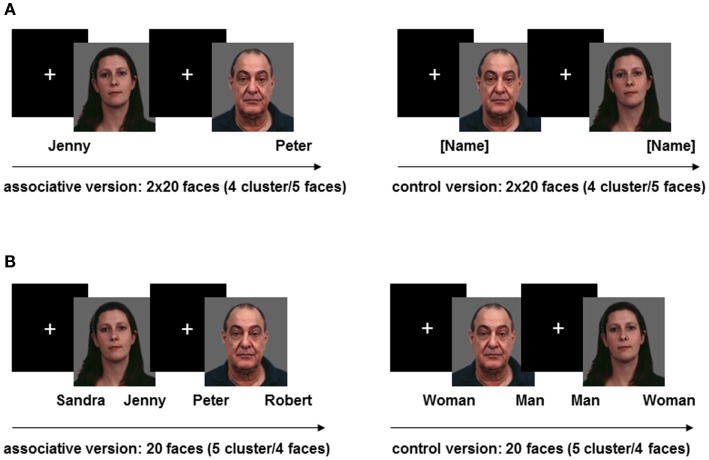
**(A)** Memory encoding task. **(B)** Memory recognition task.

The control version was previously included to allow for further ways of analyses. In order to increase the statistical power of the hemodynamic response pertaining to each event, the task was divided into 16 consecutive clusters of five faces with an alternating presentation of the two versions. Both were repeatedly announced by a 2-s instruction to either learn the names or look at the faces. Each face was shown for 5500 ms, followed by a fixation cross lasting 500 ms. Each cluster was separated by a fixation period of 13,500 ms to allow for the recovery of the hemodynamic response. During the associative cluster, subjects had to learn the association between face and name while concurrently pressing a button to indicate the side of the name (left or right) either with the middle finger (right) or the index finger (left) of their right hand. During the control cluster, participants were instructed to simply look at the faces while simultaneously indicating whether the word “name” was presented on the left or the right side of the picture. The encoding task always began with an associative cluster of the task, although the order of face presentations was pseudo-randomized between subjects. Button pressing was meant to ensure the subjects’ active engagement with the task (see Figure 1A).

#### Memory Recognition

The second task aimed to find the neural correlates of memory recognition through identification of previously learned face-name associations. To that end, all 20 faces from the encoding task were again presented twice, each either with two names (associative version) or with the German denotation of “man” and “woman” (control version) below the right and left corners of the picture. Subjects were required to decide either by button press (left or right), which of the two presented names formerly belonged to the face or whether the face is male or female. All additionally presented names without association with the current face were chosen from the pool of previously learned names. Thus, recognition of the correct name could not rely on mere novelty of the incorrect name. We presented 10 clusters of four faces according to the two versions of the task, with the task commencing with the presentation of a cluster of four faces belonging to the associative version. The order of the faces was randomized and the two task versions followed each other. Each cluster was announced by a 2-s instruction to either determine the name or the sex of the face. Each face was shown for 6500 ms followed by a fixation cross lasting 500 ms. Two clusters of faces were separated by a fixation period of 13,500 ms (see Figure 1B).

### Behavioral data analysis

Behavioral data were statistically analyzed using SPSS for Windows (version 20.0). All data are presented as means (SD) of the raw data. With regard to the neuropsychological data and the memory performance derived during fMRI, two-sample *t*-tests were used for group comparisons between patients with MCI and normally aging controls. If the variables were categorical, as in the case of some demographic enquiries, group contrasts were made on the basis of chi-square tests.

### fMRI data acquisition and analysis

Prior to scanning, all subjects were shown a training version of the two paradigms to ensure their understanding of the instructions. Then, they were placed in the MR scanner as comfortably as possible, using foam cushions to stabilize the head and minimize movement. Lenses were inserted into the goggles to ensure adequate visual acuity. The subjects’ right middle and index fingers were positioned on a response button device (LUMItouch, Lightwave Technologies, Richmond, Canada).

Neuroimaging data were acquired on a 3-T Trio MR scanner (Siemens Medical Systems, Erlangen, Germany). Echo planar imaging (EPI) sensitive to blood oxygenation level dependent (BOLD) changes (T2*, voxel size: 3.1 mm × 3.1 mm × 3.1 mm, matrix size: 64 × 64, field of view: 200 mm × 200 mm, 38 slices (AC-PC), 0.3 mm gap, TR: 2200 ms, TE: 30 ms, flip angle: 77°, volumes: 333 (encoding), 200 (recognition), total duration both tasks: 19.49 min.) was used for functional measurements. Anatomical images were obtained subsequently (3D T1*-weighted image: TE: 3.03; TR: 2300 ms; FOV = 256 mm × 256 mm; number of sagittal slices = 176; voxel size 1 mm × 1 mm × 1 mm).

For data analysis, SPM8 (Wellcome Department of Cognitive Neurology, London) was used. All following descriptions of statistical analyses apply to both tasks.

After discarding the first and last five volumes, all volumes were realigned to the remaining first volume of the time series in order to correct for head movement. Functional images were co-registered with the T1-weighted anatomical reference and transformed to Montreal Neurologic Institute (MNI) space. Normalized images were spatially smoothed using an 8-mm FWHM Gaussian kernel. As data analyses were based only on successful (hits) and unsuccessful (misses) memory performance, the individual onset times of hits and misses during associative versions of both paradigms were calculated separately. In order to define the encoding activity, the onset time of each encoded face/word combination was calculated. All correctly encoded pictures were included in the analysis of successful memory performance (successful encoding and successful recognition). Likewise, the incorrectly encoded pictures were included in the analysis of unsuccessful memory performance. Items without responses (omissions) were not taken into account due to the uncertainty as to whether or not the participants encoded or recognized them. However, as evident in Table 3, the number of omissions was extremely low, leading us to assume that the influence of omissions was not significant.

Encoding performance was based on subsequent recognition performance, assuming that successful recognition needs successful encoding. On the single subject level, individual movement parameters were used as covariates. In order to account for serial correlations in the fMRI time series, an autoregressive AR(1) model was applied. Furthermore, 128 s high-pass filtering attenuated all low-frequency signals. On a group-dependent level, a flexible factorial general linear model (GLM) was used, including the two factors group (two levels) and memory performance (two levels). Unless otherwise stated, the significance level for all main effects of the imaging data was set to *p* < 0.05 family-wise error (or FWE) corrected at the cluster level, using a cluster-defining threshold of *p* < 0.001 at the voxel level.

Contrast estimates of the group × performance interaction effect were derived using a threshold of *p* < 0.0005 (uncorrected) and a cluster extend threshold of 20 voxels in order to protect against false positives. The anatomical localization of significant clusters was based on a probabilistic cytoarchitectonic map (Eickhoff et al., 2005). For the purposes of further investigation of parameter estimates, beta values of the mean activity of a 5 mm sphere around the peak voxel were extracted for significant areas and analyzed by means of SPSS for Windows Software Package (version 20.0). Differences between parameter estimates within the interaction effect were analyzed using two-sample *t*-tests. Corollary, correlational analyses were used in order to validate a relation between selective attention (quotient of TMT-B/TMT-A) and three parameters of memory functioning (number of hits during recognition, mean activity of the parahippocampal gyrus during unsuccessful encoding, and mean activity of inferior temporal cortex during unsuccessful recognition) in patients with MCI and healthy controls separately. The Bonferroni-corrected level of significance was adapted to *p* < 0.017. The analyses regarding recognition data and correlations only comprised 18 instead of 19 patient datasets, as one patient was observed to have beta values deviating between 2.8 and 4.1 SD from the mean activity in more than half of all significant areas.

## Results

### Demographics, neuropsychology, and recognition performance

Groups did not significantly differ with regard to age, education, intelligence (MWT-B), depressive symptoms (BDI), activities of daily living (B-ADL), sex ratio, and presence of hypertension as a high-risk factor for vascular dementia. As regards neuropsychological data, patients performed worse than controls in several subtests (please refer to Table 2). The behavioral performance with reference to the fMRI paradigm did not differ significantly between groups, as both groups had nearly the same number of hits (and misses, respectively) and similar reaction times to the face stimuli (see Table 3).

**Table 2:** **Neuropsychological data**.

Test	Controls	*n*	MCI	*n*	*t*	df	*p*-Value
VLMT: total immediate recall^a^	52.78 (9.21)	27	42.95 (8.79)	19	3.63	44	0.001
VLMT: recall	11.15 (8.47)	27	8.47 (3.10)	19	3.241	44	0.002
VLMT: lost words	2.04 (1.58)	27	3.00 (2.19)	19	−1.74	44	0.09
VLMT: right recognition	13.85 (1.85)	27	13.47 (1.22)	19	0.78	44	0.441
VLMT: recognition residuals	12.48 (2.23)	27	10.68 (3.73)	19	2.05	44	0.047
WAIS: digit span forward	7.59 (1.37)	27	7.42 (2.19)	19	0.33	44	0.745
WAIS: digit span backward	6.74 (1.26)	27	5.84 (1.21)	19	2.42	44	0.02
Benton: correct drawings^b^	6.78 (1.34)	27	5.61 (1.69)	18	2.58	43	0.013
Benton: number of mistakes	4.15 (1.77)	27	7.22 (3.19)	18	−4.15	43	0.0
RWT: semantic fluency	24.48 (5.02)	27	21.83 (7.78)	18	1.39	43	0.172
RWT: semantic flexibility	16.33 (4.12)	27	13.06 (5.43)	18	2.3	43	0.026
RWT: phonological fluency	15.00 (2.57)	27	13.61 (2.48)	18	1.8	43	0.079
RWT: phonological flexibility	13.56 (3.14)	27	11.61 (4.09)	18	1.8	43	0.079
TMT: part A^c^	34.44 (12.38)	27	52.00 (21.05)	19	−3.56	44	0.001
TMT: part B	75.15 (28.32)	27	116.68 (68.65)	19	−2.83	44	0.007
Tower of London	16.26 (1.75)	27	16.05 (2.07)	19	0.37	44	0.716
MMSE-score	29.22 (0.75)	27	28.78 (0.94)	18	1.76	43	0.086
SKT-score	1.11 (1.28)	27	2.26 (2.58)	19	−2	44	0.051
TFDD-score^d^	44.78 (2.21)	27	42.42 (3.22)	19	2.95	44	0.005

*Values are presented as means (SD)*.

*p ≤ 0.01 is considered significant according to Bonferroni-Holm corrections for multiple testing.

*Degrees of freedom (df) have decimals when the Levene-test for the equality of variances is significant*.

*^a^German version of the California Verbal learning test*.

*^b^Benton Visual Retention test, German version*.

*^c^Trail-Making Test*.

*^d^Test zur Frueherkennung von Demenzen mit Depressionsabgrenzung*.

**Table 3:** **fMRI recognition performance**.

	Controls (*n* = 27)	MCI (*n* = 19)
Hits	15,11 (2.1)	14.16 (2.4)
Misses	4.85 (2.2)	5.79 (2.4)
Omissions	0.04 (0.2)	0.05 (0.2)
Percent correct	75.56 (10.7)	71.05 (11.9)
Reaction time hits (ms)	2537.23 (514.0)	2643.98 (465.5)
Reaction time misses (ms)	1995.01 (963.5)	2828.29 (596.2)

*Values are presented as means (SD)*.

*There are no statistical differences with *p* < 0.05 considered significant*.

### fMRI results

#### Memory encoding versus recognition

**Figure 2:**
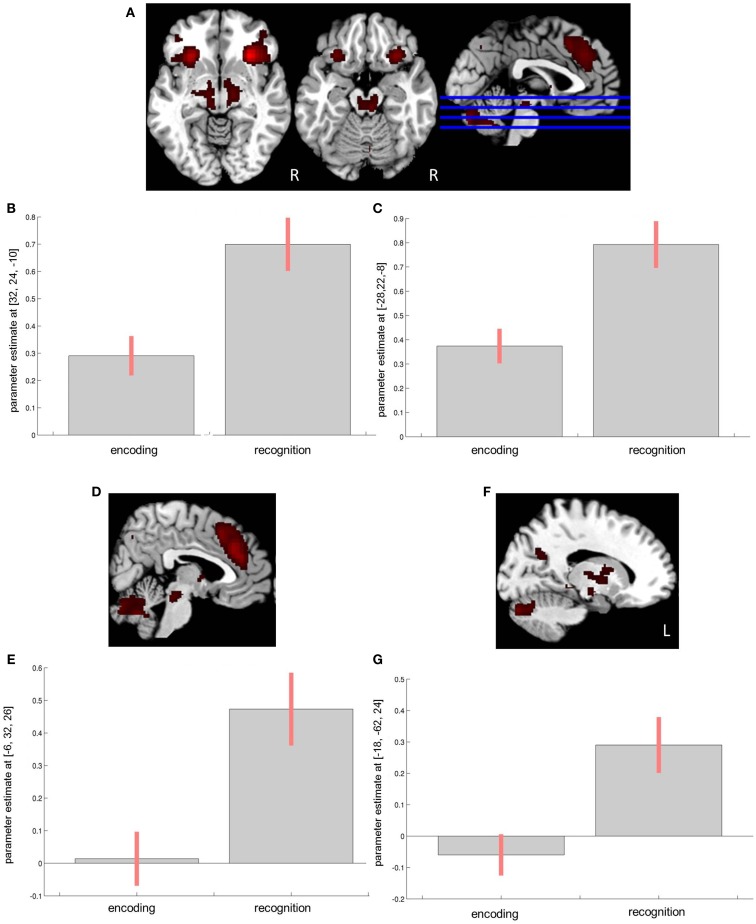
**Encoding × recognition interaction in the control group**. **(A)** Effects in the bilateral inferior frontal gyrus/insula region. **(B)** Corres-ponding parameter estimates from the right insula. **(C)** Corresponding parameter estimates from the left insula. **(D)** Effects in the anterior cingulate cortex (ACC). **(E)** Corresponding parameter estimates from the ACC. **(F)** Effects in the left cuneus. **(G)** Corresponding parameter estimates from the left cuneus.

In the control group, at *p* < 0.001, interaction between encoding and recognition was seen in the left insula (*F* = 45.57; MNI *x* = −28, *y* = 22, *z* = −8; 947 voxels), the right inferior frontal gyrus/insula (*F* = 60.97; MNI *x* = 32, *y* = 24, *z* = −10; 2012 voxels), the orbitofrontal cortex (OFC) including the ACC (*F* = 36.71; MNI *x* = 6, *y* = 32, *z* = 26; 2461 voxels), the right basal ganglia (*F* = 22.63; MNI *x* = 14, *y* = 8, *z* = 8; 2008 voxels), the left cerebellum (*F* = 30.72; MNI *x* = −8, *y* = −84, *z* = −34; 1377 voxels), the left middle orbital gyrus (*F* = 23.50; MNI *x* = −36, *y* = 52, *z* = −2; 524 voxels), the left cuneus/precuneus region(*F* = 17.32; MNI *x* = −18, *y* = −64, *z* = 24; 177 voxels), the right middle frontal gyrus (*F* = 18.85; MNI *x* = 36, *y* = 26, *z* = 36; 151 voxels), and the right middle orbital gyrus (*F* = 11.30; MNI *x* = 26, *y* = 52, *z* = −7; 40 voxels) (Figure 2).

Among patients, the effects were seen in the bilateral insula/inferior frontal gyrus (*F* = 24.25; MNI *x* = −26, *y* = 24, *z* = −8; 1475 voxels and *F* = 27.64; MNI *x* = 30, *y* = 24, *z* = −12; 1101 voxels for the left and right insula, respectively), the left dorsal ACC/supplemental motor area (*F* = 14.86; MNI *x* = −6, *y* = 20, *z* = 44; 939 voxels), the left precuneus/cuneus region (*F* = 13.96; MNI *x* = −12, *y* = −64, *z* = 32; 827 voxels), the left thalamus (*F* = 21.79; MNI *x* = −8, *y* = −18, *z* = −14; 638 voxels), the bilateral cerebellum (*F* = 13.00; MNI *x* = 12, *y* = −84, *z* = −30; 553 voxels), the right angular gyrus (*F* = 13.16; MNI *x* = 42, *y* = −48, *z* = 36; 224 voxels), the right middle frontal gyrus (*F* = 9.73; MNI *x* = 42, *y* = 54, *z* = 4; 58 voxels), and the right inferior temporal gyrus (*F* = 10.30; MNI *x* = 58, *y* = −26, *z* = −26; 20 voxels).

The encoding × recognition × group interaction at *p* < 0.05 uncorrected was seen only in the rostral ACC (*F* = 6.91; MNI *x* = 4, *y* = 38, *z* = 22; 106 voxels).

#### Memory encoding

In the patient group, at *p* < 0.05 family-wise error-corrected at the cluster level, stronger involvement of the OFC (including the rostral ACC), the bilateral precuneus, the left middle temporal gyrus, and the right caudate nucleus was seen during successful as compared to unsuccessful encoding (for additional areas significant at *p* < 0.001 uncorrected, see Table S1 and Figure S1 in Supplementary Materials). The opposite contrast in the patient group was not significant.

To demonstrate the common effects of successful versus unsuccessful encoding in both groups, we included the two *t*-contrasts (successful encoding > unsuccessful encoding in controls and successful encoding > unsuccessful encoding in patients, each at *P* < 0.05 uncorrected with a cluster extent of >40 voxels) into a conjunction analysis, which revealed stronger involvement of the left insula (*Z* = 3.57; MNI *x* = −44, *y* = 12, *z* = −10; 132 voxels), the right insula (*Z* = 2.34; MNI *x* = 32, *y* = 18, *z* = −6; 74 voxels), the OFC including the rostral ACC (*Z* = 2.23; MNI *x* = −2, *y* = 46, *z* = 0; 90 voxels), and the left hippocampus (*Z* = 2.40; MNI *x* = −28, *y* = −10, *z* = −22; 42 voxels) during successful encoding. As demonstrated by the parameter estimates, the rostral ACC and the left insula showed stronger deactivation during unsuccessful encoding in both groups. In the left hippocampus, on the other hand, in both groups, the effect was stronger during successful encoding (see Figure S2 in Supplementary Materials).

**Figure 3:**
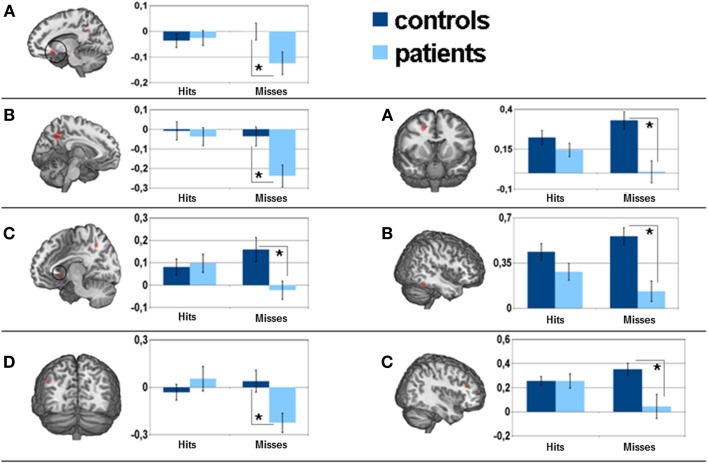
**Left side**: parameter estimates of the encoding-success × group interaction effect and corresponding 5 mm sphere mean activity for separate factors: **(A)** left orbitofrontal cortex, **(B)** left precuneus, **(C)** left parahippocampal gyrus, **(D)** left angular gyrus. **Right side**: neural correlates of the recognition-success × group interaction effect and corresponding 5 mm sphere mean activity for separate factors: **(A)** right middle frontal gyrus, **(B)** right inferior temporal cortex, **(C)** right dorsolateral prefrontal cortex. *is considered significant at **(A)** *p* = 0.025, **(B)** *p* = 0.005, **(C)** *p* = 0.008, **(D)** *p* = 0.006, respectively **(A)** *p* = 0.001. **(B)** *p* = 0.00, **(C)** *p* = 0.003.

Significant areas of the group × encoding-success interaction were observed in frontal areas [OFC, superior frontal, anterior cingulate cortex (ACC)], parietal regions (precuneus, angular gyrus, intraparietal sulcus), subcortical regions (caudate nucleus) and temporal areas (entorhinal cortex, middle temporal cortex) (see Table 3). Statistical analysis of parameter estimates in these regions neither yielded significant differences regarding activity between hits and misses in controls, nor significant differences between controls and patients with regard to hits. However, the activity in the patient group varied significantly with encoding-success. In more detail, patients had significantly lower activity in almost all areas during unsuccessful encoding as compared to successful encoding. With reference to the comparison of activity during unsuccessful encoding between patients and controls, patients showed significantly lower activity in the following areas: bilateral OFC, left precuneus, right insular cortex, left entorhinal cortex/hippocampus, and left angular gyrus. There was no brain area showing significantly higher activation during unsuccessful encoding in patients as compared to successful encoding in patients or unsuccessful encoding in controls. Please see Figure 3 for visualization of clusters and corresponding parameter estimates and Table 4 for corresponding means and standard errors.

**Table 4:** **Activation peaks of the group × memory-success interaction effect**.

Anatomical location	*x*	*y*	*z*	*F*-value	Number of voxels
**Encoding**
Left orbitofrontal cortex*	−14	20	−16	25.44	59
Right orbitofrontal cortex*	8	52	−16	18.46	29
Right middle orbital gyrus	4	18	−4	20.04	109
Right superior frontal gyrus	34	58	20	24.25	246
Left precuneus*	−6	−58	36	23.67	560
Right insular cortex	26	−30	26	21.22	46
Right insular cortex*	30	0	24	19.73	37
Right anterior cingulate cortex	14	34	−6	20.74	53
Left entorhinal cortex*	−14	−26	−14	20.48	25
Right caudate nucleus	14	4	12	19.81	42
Right intraparietal sulcus	28	−56	30	18.90	37
Right middle temporal cortex	38	−60	26	15.82	22
Left angular gyrus*	−48	−66	36	15.81	30
**Recognition**
Right middle frontal gyrus*	10	16	46	21.58	105
Left supramarginal gyrus	−32	−30	22	21.50	36
Right inferior temporal cortex*	48	−48	−22	19.30	50
Right dorsolateral prefrontal cortex*	38	34	22	19.29	37
Left thalamus*	−26	−26	−4	19.19	23
Left cerebellum	−44	−60	−22	18.73	43
Right anterior cingulate cortex*	20	−2	36	16.00	23

*Coordinates are given in MNI-space*.

*An asterisk indicates areas with differences between groups regarding false memory performance*.

#### Memory recognition

At *p* < 0.05 family-wise error-corrected at the cluster level, unsuccessful compared to successful recognition in the control group yielded significantly higher levels of activity in the occipital areas (including the extrastriate visual cortex), the dorsal prefrontal cortex (DLPFC), the dorsal anterior cingulate cortex (dACC), and the inferior temporal cortex (for other areas significant at *p* < 0.001 uncorrected, see Table S1 in Supplementary Material). The opposite contrast did not show any differences. In patients, no areas were identified with significantly higher (or lower) activity during unsuccessful, compared to successful, recognition.

The group × recognition success interaction showed significant effects in the frontal areas (middle frontal gyrus, dorsolateral prefrontal, ACC), the parietal regions (supramarginal gyrus), the temporal areas (inferior temporal cortex), the thalamus, and the cerebellum.

A comparison between groups based on unsuccessful recognition revealed significantly lower activity in patients in almost all affected areas (see Table 3). For visualization of clusters and corresponding parameter estimates, please see Figure 3.

### Corollary correlational analyses

In patients with MCI, there was a significant negative correlation between selective attention and the number of hits during recognition (*r* = −0.575, *p* = 0.013). We also found a significant correlation between memory function (VLMT: total immediate recall) and the number of hits during recognition (*r* = 0.396, *p* = 0.041). However, selective attention and memory did not significantly correlate either with the mean activity of the entorhinal cortex during unsuccessful encoding (*r* = 0.022, *p* = 0.930) or with the mean activity of the inferior temporal cortex during unsuccessful recognition (*r* = 0.037, *p* = 0.883).

In healthy controls, there was no significant correlation between selective attention and, on the one hand, the number of hits during recognition (*r* = −0.085, *p* = 0.674), and, on the other, the mean activity of the entorhinal cortex during unsuccessful encoding (*r* = −0.031, *p* = 0.879) and the mean activity of the inferior temporal cortex during unsuccessful recognition (*r* = 0.079, *p* = 0.694). No significant association between memory function (VLMT: total immediate recall) and the number of hits during recognition was observed either.

## Discussion

The purpose of the current study was to investigate potential differences between patients with stable amnestic MCI and healthy elderly controls with regard to the neural correlates of successful versus unsuccessful encoding and recognition.

In the control group, we observed decreased activity during encoding and an increase during retrieval in, among other regions, the medial prefrontal areas (including ACC), the bilateral insula/inferior frontal cortex and the occipital areas (e.g., cuneus). Young, healthy subjects tend to exhibit a decrease in activity during encoding and an increase during retrieval (also known as encoding/revival “flip”) (Vannini et al., 2013). Negative encoding activity (or negative subsequent memory effects) is often deemed beneficial to successful performance (Daselaar et al., 2004; Uncapher et al., 2011; de Chastelaine and Rugg, 2014) and is linked to activity in the default network which decreases during tasks demanding attention to external stimuli and novel events (Greicius et al., 2009). The impaired ability to modulate activity in the default network regions is associated with age, greater amyloid burden, and worse memory performance (Vannini et al., 2013). As our results demonstrate, unsuccessful (compared to successful) encoding in particular is associated with decreased activity in the rostral ACC and the left insula in both patients and healthy individuals. On the other hand, the left hippocampus was more strongly involved during successful encoding in both groups, presumably reflecting successful memory processes. This observation is consistent with the results of previous studies (e.g., Miller et al., 2008).

Given that our patient group comprised stable MCI patients, a considerable part of the group might never develop full-blown dementia. The fact that patients and controls did not differ with regard to successful encoding memory performance, and that the effect on activation during the unsuccessful condition did not correspond to increases in the number of false alarms, might indicate that this group of stable amnestic MCI patients (compared to healthy aging controls) still had equal resources to correctly encode and recognize memory-related stimulus material.

However, in the patient group, between unsuccessful (as compared to successful) encoding trials, we observed differences in negative encoding activity with patients showing further decreased activity in the frontal and parietal regions. Among the controls, on the other hand, the differences between the two types of encoding trials were less prominent.

Successful encoding is dependent on the effective interplay of temporal, parietal, and frontal regions (Chun and Turk-Browne, 2007; Miller et al., 2008). In the current group of patients with stable amnestic MCI, these particular regions showed task-related deactivation compared to controls for poorly remembered associations (unsuccessful encoding). While the entorhinal cortex in the medial temporal lobe is particularly linked to memory processes, the frontoparietal network is related to visual attention (Corbetta, 1998). Thus, an emerging dysfunction in the attentional memory system in patients with stable amnestic MCI may be a possible explanation of our findings. This assumption is further corroborated by the significant negative correlation, we observed between a parameter for selective attention and the number of hits during recognition. As limited attentional resources are shared by information processing and memory, top-down attention is necessary for the transmission of only highly salient information to the memory-related areas (Bunting et al., 2008; Sestieri et al., 2010; Uncapher et al., 2011). During unsuccessful encoding, a temporary under-recruitment of frontal, parietal, or both areas might prevent goal-based selection of interfering stimuli, resulting in an uncontrolled propagation of the to-be-encoded material to memory-related areas.

Unsuccessful, compared to successful, recognition in the control group led to higher activity in the extrastriate visual cortex, the dorsolateral prefrontal cortex (DLPFC), and the dACC. A comparison between the groups based on unsuccessful recognition showed lower activity of those areas in patients. Thus, false recognition led to under-recruitment of the frontal areas and the inferior temporal cortex in patients, although, again, without any effect on behavioral performance. It has been suggested that MCI patients might display decreased and non-compensatory brain activity with the progression of dementia (Sperling, 2007; Dickerson and Sperling, 2008; Machulda et al., 2009). Proposing a deficit in selective attention, Stevens et al. (2008) have observed that memory performance declines due to impaired inhibition of irrelevant information during encoding. Selective attention is particularly affected in AD, leading to ineffective search and inhibition mechanisms (Foldi et al., 2002). According to the findings of Silveri et al. (2007), lower scores on selective attention tasks in patients with MCI predict the development of dementia later in life. Similarly, Shimada et al. (2012) have found attention tasks to be useful predictors of atrophy in the medial temporal lobe and entorhinal cortex. Some studies have even proposed a so-called anatomo-functional syndrome in AD, hypothesizing that deficits in attention may lead to memory problems, corroborating the notion of a common deficient mechanism in MCI (Silveri et al., 2007). Collectively, a dysfunctional interplay between the top-down control of frontal areas and face perception of the inferior temporal cortex is purported to be responsible for false recognition in patients with MCI. Their memory failures, therefore, may be caused subsequently during consolidation or recognition. In fact, false recognition in the control group yielded significantly higher activity in DLPFC, dorsal ACC, and inferior temporal cortex than correct recognition. Based on the observations that the DLPFC is necessary for successful retrieval (Rugg et al., 2002; Murray and Ranganath, 2007; Stevens et al., 2008), the inferior temporal cortex is associated with familiar face perception (Schweinberger et al., 2002), and the ACC with decision making and conflict monitoring (Bunting et al., 2008; Chechko et al., 2012, 2013), it may be suggested that during difficult trials, subjects try to allocate extra resources to search-related retrieval processes.

The fact that the analysis involved an unequal distribution of successful and unsuccessful trials may be deemed a limitation of our study. As the number of unsuccessful trials constituted only about 25% of all events, the difference between variances might have prevented weaker activity changes to become significant. Furthermore, encoding performance was linked to subsequent recognition performance based on the assumption that successful recognition needs successful encoding. Because of the repetition of the face/name pairs, it was also not possible to pinpoint exactly during which trial the item was correctly encoded. However, unsuccessful recognition does not automatically imply that encoding too has been unsuccessful. In fact, despite successful encoding, memory may fail due to unsuccessful consolidation or recognition. Moreover, in the associative version, each face/name was shown twice. Although the number of repetitions was relatively low, it could still be considered as one of the limitations of the paper. While we cannot, indeed, entirely exclude the possibility that repetition suppression/enhancement might have occurred in our experiment, our methodology would not be conducive to studying this phenomenon due to complete and large temporal separation of initial presentations and repetitions (and the consequential low-frequency of any repetition effects). Finally, the omissions were not included in the model owing to their small number and the fact that we could not determine if they represented patients’ failure to encode or recognized items.

In summary, despite equal memory performance in the fMRI paradigm, during unsuccessful encoding and recognition, patients were found to hypoactivate particularly the frontal, parietal, and temporal areas of the brain. Based on the results of previous research, we proposed that a temporary dysfunction of the top-down control of the frontal or parietal or both areas might result in non-selective propagation of task-related information to memory. The lack of differences observed in behavioral performance is in contrast to previous findings, suggesting that patients with MCI either hyperactivate particular brain areas in order to compensate for their cognitive difficulties or hypoactivate them as a matter of malfunction (Sperling et al., 2003; Dickerson et al., 2005; Celone et al., 2006; Hämäläinen et al., 2007; Clément and Belleville, 2010; O’Brien et al., 2010; Kim et al., 2012). Unlike our study, most previous studies have failed to make a clear distinction between successful and unsuccessful memory performance, basing their analyses purely on encoding or recognition. Thus, their findings may have resulted from pooling two sides of the same cognitive process, which, as our results now suggest, may actually have been affected differentially by MCI. The fact that in our study patients and controls did not differ with regard to successful and unsuccessful memory performance indicates that this group of stable amnestic MCI patients still had enough recourses, suggesting that the observed deactivation in the default network regions might very well have been a compensatory mechanism. The lack of difference between MCIs and controls in the memory task (during successful und unsuccessful memory performance) might also have been due to the fact that most of the stable MCI patients would not develop AD. As the current sample of patients included only those with multiple amnestic MCI, with attention being more strongly affected in comparison to other domains, the results are likely to be evaluated in terms of attentional and memory deficits. Consequently, the findings cannot be automatically extended to other MCI groups.

Future research should put more emphasis on the investigation of failure mechanisms during unsuccessful cognitive performance in elderly patients in order to obtain a more thorough understanding of preclinical dementia processes and further potential risk factors.

## Conflict of Interest Statement

The authors declare that the research was conducted in the absence of any commercial or financial relationships that could be construed as a potential conflict of interest.

## Supplementary Material

The Supplementary Material for this article can be found online at http://www.frontiersin.org/Journal/10.3389/fnagi.2014.00201/abstract

## References

[B1] Bondi M. W. Houston W. S. Eyler L. T. Brown G. G. (2005). fMRI evidence of compensatory mechanisms in older adults at genetic risk for Alzheimer disease. Neurology 64, 501–508 10.1212/01.wnl.0000150885.00929.7e 15699382 PMC1761695

[B2] Bunting M. F. Cowan N. Colflesh G. H. (2008). The deployment of attention in short-term memory tasks: trade-offs between immediate and delayed deployment. Mem. Cognit. 36, 799–812 10.3758/mc.36.4.799 18604962 PMC2667108

[B3] Busse A. Hensel A. Gühne U. Angermeyer M. C. Riedel-Heller S. G. (2006). Mild cognitive impairment: long-term course of four clinicl subtypes. Neurology 67, 2176–2185 10.1212/01.wnl.0000249117.23318.e1 17190940

[B4] Celone K. A. Calhoun V. D. Dickerson B. C. Atri A. Chua E. F. Miller S. L. (2006). Alterations in memory networks in mild cognitive impairment and Alzheimer’s disease: an independent component analysis. J. Neurosci. 26, 10222–10231 10.1523/jneurosci.2250-06.2006 17021177 PMC6674636

[B5] Chechko N. Augustin M. Zvyagintsev M. Schneider F. Habel U. Kellermann T. (2013). Brain circuitries involved in emotional interference task in major depression disorder. J. Affect. Disord. 149, 136–145 10.1016/j.jad.2013.01.013 23394712

[B6] Chechko N. Kellermann T. Zvyagintsev M. Augustin M. Schneider F. Habel U. (2012). Brain circuitries involved in semantic interference by demands of emotional and non-emotional distractors. PLoS ONE 7:e38155 10.1371/journal.pone.0038155 22666470 PMC3362560

[B7] Chun M. M. Turk-Browne N. B. (2007). Interactions between attention and memory. Curr. Opin. Neurobiol. 17, 177–184 10.1016/j.conb.2007.03.005 17379501

[B8] Clément F. Belleville S. (2010). Compensation and disease severity on the memory-related activations in mild cognitive impairment. Biol. Psychiatry 68, 894–902 10.1016/j.biopsych.2010.02.004 20359695

[B9] Corbetta M. (1998). Frontoparietal cortical networks for directing attention and the eye to visual locations: identical, independent, or overlapping neural systems? Proc. Natl. Acad. Sci. U.S.A. 95, 831–838 10.1073/pnas.95.3.831 9448248 PMC33805

[B10] Daly E. Zaitchik D. Copeland M. Schmahmann J. Gunther J. Albert M. (2000). Predicting conversion to Alzheimer disease using standardized clinical information. Arch. Neurol. 57, 675–680 10.1001/archneur.57.5.675 10815133

[B11] Daselaar S. M. Prince S. E. Cabeza R. (2004). When less means more: deactivations during encoding that predict subsequent memory. Neuroimage 23, 921–927 10.1016/j.neuroimage.2004.07.031 15528092

[B12] DeCarli C. (2003). Mild cognitive impairment: prevalence, prognosis, aetiology, and treatment. Lancet Neurol. 2, 15–21 10.1016/S1474-4422(03)00262-X 12849297

[B13] de Chastelaine M. Rugg M. D. (2014). The relationship between task-related and subsequent memory effects. Hum. Brain Mapp. 35, 3687–3700 10.1002/hbm.22430 24615858 PMC4107062

[B14] Dickerson B. C. Salat D. H. Bates J. F. Atiya M. Killiany R. J. Greve D. N. (2004). Medial temporal lobe function and structure in mild cognitive impairment. Ann. Neurol. 56, 27–35 10.1002/ana.20163 15236399 PMC4335689

[B15] Dickerson B. C. Salat D. H. Greve D. N. Chua E. F. Rand-Giovannetti E. Rentz D. M. (2005). Increased hippocampal activation in mild cognitive impairment compared to normal aging and AD. Neurology 65, 404–411 10.1212/01.wnl.0000171450.97464.49 16087905 PMC4335677

[B16] Dickerson B. C. Sperling R. A. (2008). Functional abnormalities of the medial temporal lobe memory system in mild cognitive impairment and Alzheimer’s disease: insights from functional MRI studies. Neuropsychologia 46, 1624–1635 10.1016/j.neuropsychologia.2007.11.030 18206188 PMC2760288

[B17] Drexler E. I. Voss B. Amunts K. Schneider F. Habel U. (2013). Mild cognitive impairment: advantages of a comprehensive neuropsychological assessment. Curr. Alzheimer Res. 10, 1098–1106 10.2174/15672050113109990014 22746244

[B18] Eickhoff S. B. Stephan K. E. Mohlberg H. Grefkes C. Fink G. R. Amunts K. (2005). A new SPM toolbox for combining probabilistic cytoarchitectonic maps and functional imaging data. Neuroimage 25, 1325–1335 10.1016/j.neuroimage.2004.12.034 15850749

[B19] Foldi N. S. Lobosco J. J. Schaefer L. A. (2002). The effect of attentional dysfunction in Alzheimer’s disease: theoretical and practical implications. Semin. Speech Lang. 23, 139–150 10.1055/s-2002-24990 11951174

[B20] Gauthier S. Reisberg B. Zaudig M. Petersen R. C. Ritchie K. Broich K. (2004). Mild cognitive impairment. Lancet 367, 1262–1270 10.1016/S0140-6736(06)68542-5 16631882

[B21] Greicius M. D. Supekar K. Menon V. Dougherty R. F. (2009). Resting-state functional connectivity reflects structural connectivity in the default mode network. Cereb. Cortex 19, 72–78 10.1093/cercor/bhn059 18403396 PMC2605172

[B22] Hämäläinen A. Pihlajamäki M. Tanila H. Hänninen T. Niskanen E. Tervo S. (2007). Increased fMRI responses during encoding in mild cognitive impairment. Neurobiol. Aging 28, 1889–1903 10.1016/j.neurobiolaging.2006.08.008 16997428

[B23] Heun R. Freymann K. Erb M. Leube D. T. Jessen F. Kircher T. T. (2007). Mild cognitive impairment (MCI) and actual retrieval performance affect cerebral activation in the elderly. Neurobiol. Aging 28, 404–413 10.1016/j.neurobiolaging.2006.01.012 16530885

[B24] Johnson S. C. Schmitz T. W. Moritz C. H. Meyerand M. E. Rowley H. A. Alexander A. L. (2006). Activation of brain regions vulnerable to Alzheimer’s disease: the effect of mild cognitive impairment. Neurobiol. Aging 27, 1604–1612 10.1016/j.neurobiolaging.2005.09.017 16226349 PMC2627778

[B25] Kim M.-J. Lee K.-M. Son Y.-D. Jeon H.-A. Kim Y.-B. Cho Z.-H. (2012). Increased basal forebrain metabolism in mild cognitive impairment: an evidence for brain reserve in incipient dementia. J. Alzheimer Dis. 32, 927–938 10.3233/jad-2012-120133 22903128

[B26] Kircher T. T. Weis S. Freymann K. Erb M. Jessen F. Grodd W. (2007). Hippocampal activation in patients with mild cognitive impairment is necessary for successful memory encoding. J. Neurol. Neurosurg. Psychiatry 78, 812–818 10.1136/jnnp.2006.104877 17287238 PMC2117738

[B27] Machulda M. M. Senjem M. L. Weigand S. D. Smith G. E. Ivnik R. J. Boeve B. F. (2009). Functional magnetic resonance imaging changes in amnestic and nonamnestic mild cognitive impairment during encoding and recognition tasks. J. Int. Neuropsychol. Soc. 15, 372–382 10.1017/S1355617709090523 19402923 PMC2762430

[B28] Miller S. L. Celone K. Depeau K. Diamond E. Dickerson B. C. Rentz D. (2008). Age-related memory impairment associated with loss of parietal deactivation but preserved hippocampal activation. Proc. Natl. Acad. Sci. U.S.A. 105, 2181–2186 10.1073/pnas.0706818105 18238903 PMC2538895

[B29] Murray L. J. Ranganath C. (2007). The dorsolateral prefrontal cortex contributes to successful relational memory encoding. J. Neurosci. 27, 5515–5522 10.1523/jneurosci.0406-07.2007 17507573 PMC6672342

[B30] O’Brien J. L. O’Keefe K. M. Laviolette P. S. Deluca A. N. Blacker D. Dickerson B. C. (2010). Longitudinal fMRI in elderly reveals loss of hippocampal activation with clinical decline. Neurology 74, 1969–1976 10.1212/WNL.0b013e3181e3966e 20463288 PMC2905893

[B31] Oldfield R. C. (1971). The assessment and analysis of handedness: the Edinburgh inventory. Neuropsychologia 9, 97–113 10.1016/0028-3932(71)90067-4 5146491

[B32] Petersen R. C. Negash S. (2008). Mild cognitive impairment: an overview. CNS Spectr. 13, 45–53 18204414 10.1017/s1092852900016151

[B33] Petrella J. R. Krishnan S. Slavin M. J. Tran T.-T. T. Murty L. Doraiswamy P. M. (2006). Mild cognitive impairment: evaluation with 4-T functional MR imaging. Radiology 240, 177–186 10.1148/radiol.2401050739 16684919

[B34] Portet F. Ousset P. J. Visser P. J. Frisoni G. B. Nobili F. Scheltens P. (2006). Mild cognitive impairment (MCI) in medical practice: a critical review of the concept and new diagnostic procedure. Report of the MCI Working Group of the European Consortium on Alzheimer’s Disease. J. Neurol. Neurosurg. Psychiatry 77, 714–718 10.1136/jnnp.2005.085332 16549412 PMC2077456

[B35] Ries M. L. Carlsson C. M. Rowley H. A. Sager M. A. Gleason C. E. Asthana S. (2008). Magnetic resonance imaging characterization of brain structure and function in mild cognitive impairment: a review. J. Am. Geriatr. Soc. 56, 920–934 10.1111/j.1532-5415.2008.01684.x 18410325 PMC2668820

[B36] Rizzo M. Anderson S. Dawson J. Myers R. Ball K. (2000). Visual attention impairments in Alzheimer’s disease. Am. J. Ophthalmol. 130, 384–385 10.1016/S0002-9394(00)00699-1 11020439

[B37] Rugg M. D. Otten L. J. Henson R. N. A. (2002). The neural basis of episodic memory: evidence from functional neuroimaging. Philos. Trans. R. Soc. Lond. B Biol. Sci. 357, 1097–1110 10.1098/rstb.2002.1102 12217177 PMC1693015

[B38] Schweinberger S. R. Pickering E. C. Jentzsch I. Burton A. M. Kaufmann J. M. (2002). Event-related brain potential evidence for a response of inferior temporal cortex to familiar face repetitions. Cogn. Brain Res. 14, 398–409 10.1016/S0926-6410(02)00142-8 12421663

[B39] Sestieri C. Shulman G. L. Corbetta M. (2010). Attention to memory and the environment: functional specialization and dynamic competition in human posterior parietal cortex. J. Neurosci. 30, 8445–8456 10.1523/jneurosci.4719-09.2010 20573892 PMC2906749

[B40] Shimada H. Kato T. Ito K. Makizako H. Doi T. Yoshida D. (2012). Relationship between Atrophy of the medial temporal areas and cognitive functions in elderly adults with mild cognitive impairment. Eur. Neurol. 67, 168–177 10.1159/000334845 22286117

[B41] Silveri M. C. Reali G. Jenner C. Puopolo M. (2007). Attention and memory in the preclinical stage of dementia. J. Geriatr. Psychiatry Neurol. 20, 67–75 10.1177/0891988706297469 17548775

[B42] Sperling R. (2007). Functional MRI studies of associative encoding in normal aging, mild cognitive impairment, and Alzheimer’s disease. Ann. N. Y. Acad. Sci. 1097, 146–155 10.1196/annals.1379.009 17413017

[B43] Sperling R. Chua E. Cocchiarella A. Rand-Giovannetti E. Poldrack R. Schacter D. L. (2003). Putting names to faces: successful encoding of associative memories activates the anterior hippocampal formation. Neuroimage 20, 1400–1410 10.1016/S1053-8119(03)00391-4 14568509 PMC3230827

[B44] Stevens W. D. Hasher L. Chiew K. S. Grady C. L. (2008). A neural mechanism underlying memory failure in older adults. J. Neurosci. 28, 12820–12824 10.1523/jneurosci.2622-08.2008 19036975 PMC2613641

[B45] Trivedi M. A. Murphy C. M. Goetz C. Shah R. C. Gabrieli J. D. E. Whitfield-Gabrieli S. (2008). fMRI activation changes during successful episodic memory encoding and recognition in amnestic mild cognitive impairment relative to cognitively healthy older adults. Dement. Geriatr. Cogn. Disord. 26, 123–137 10.1159/000148190 18663302 PMC2760214

[B46] Uncapher M. R. Hutchinson J. B. Wagner A. D. (2011). Dissociable effects of top-down and bottom-up attention during episodic encoding. J. Neurosci. 31, 12613–12628 10.1523/jneurosci.0152-11.2011 21880922 PMC3172893

[B47] Vannini P. Hedden T. Huijbers W. Ward A. Johnson K. A. Sperling R. A. (2013). The ups and downs of the posteromedial cortex: age- and amyloid-related functional alterations of the encoding/retrieval flip in cognitively normal older adults. Cereb. Cortex 23, 1317–1328 10.1093/cercor/bhs108 22586140 PMC3643714

[B48] Winblad B. Palmer K. Kivipelto M. Jelic V. Fratiglioni L. Wahlund L. O. (2004). Mild cognitive impairment – beyond controversies, towards a consensus: report of the International Working Group on Mild Cognitive Impairment. J. Intern. Med. 256, 240–246 10.1111/j.1365-2796.2004.01380.x 15324367

