# Supplementary material for: Neural Correlates of Unsuccessful Memory Performance in MCI
Source: Front Aging Neurosci. 2014 Aug 13;6:201. doi: 10.3389/fnagi.2014.00201 (PMC4131189; doi:10.3389/fnagi.2014.00201)
Supplement: Supplementary file 1 [file Presentation1.PDF]

Supplementary Material legends

Figure S1

Areas with stronger deactivation in response to unsuccessful as compared to successful encoding in patients

A. Effects in the orbitofrontal cortex and cuneus
B. Effects in the right basal ganglia

Effects are shown $p<0.05$ family-wise error corrected at cluster level

Figure S2

Common effects of successful versus unsuccessful memory encoding in both groups (conjunction analysis)

A. Effects and the corresponding parameter estimates in the rostral ACC
B. Effects and the corresponding parameter estimates in the left hippocampus
C. Effects in the bilateral insula and corresponding parameter estimates in the left insula

Condition 1 Successful memory encoding effect in controls

Condition 2 Unsuccessful memory encoding effect in controls

Condition 3 Successful memory encoding effect in patients

Condition3 Unsuccessful memory encoding effect in patients

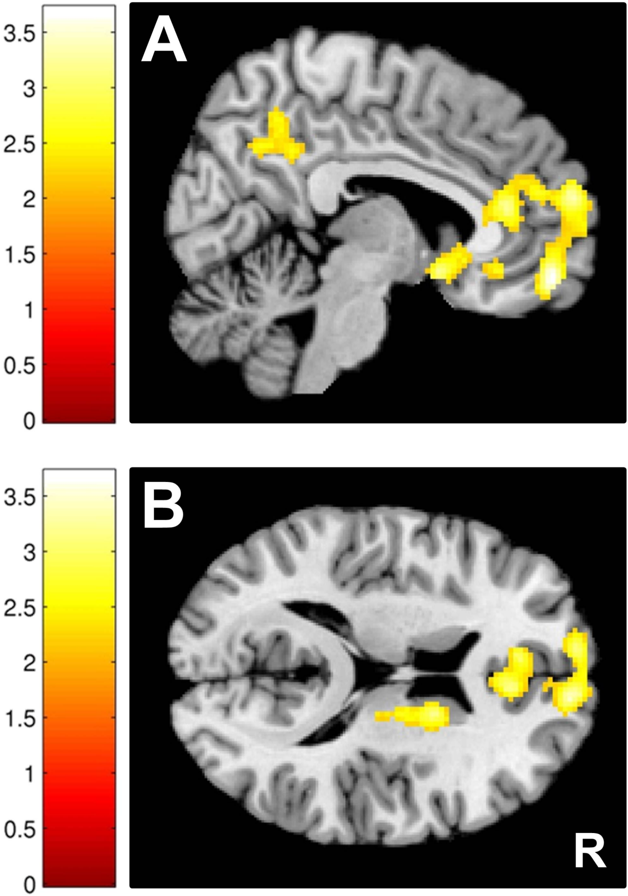

3.5
3
2.5
2
1.5
1
0.5
0
A
3.5
3
2.5
2
1.5
1
0.5
0
B
R

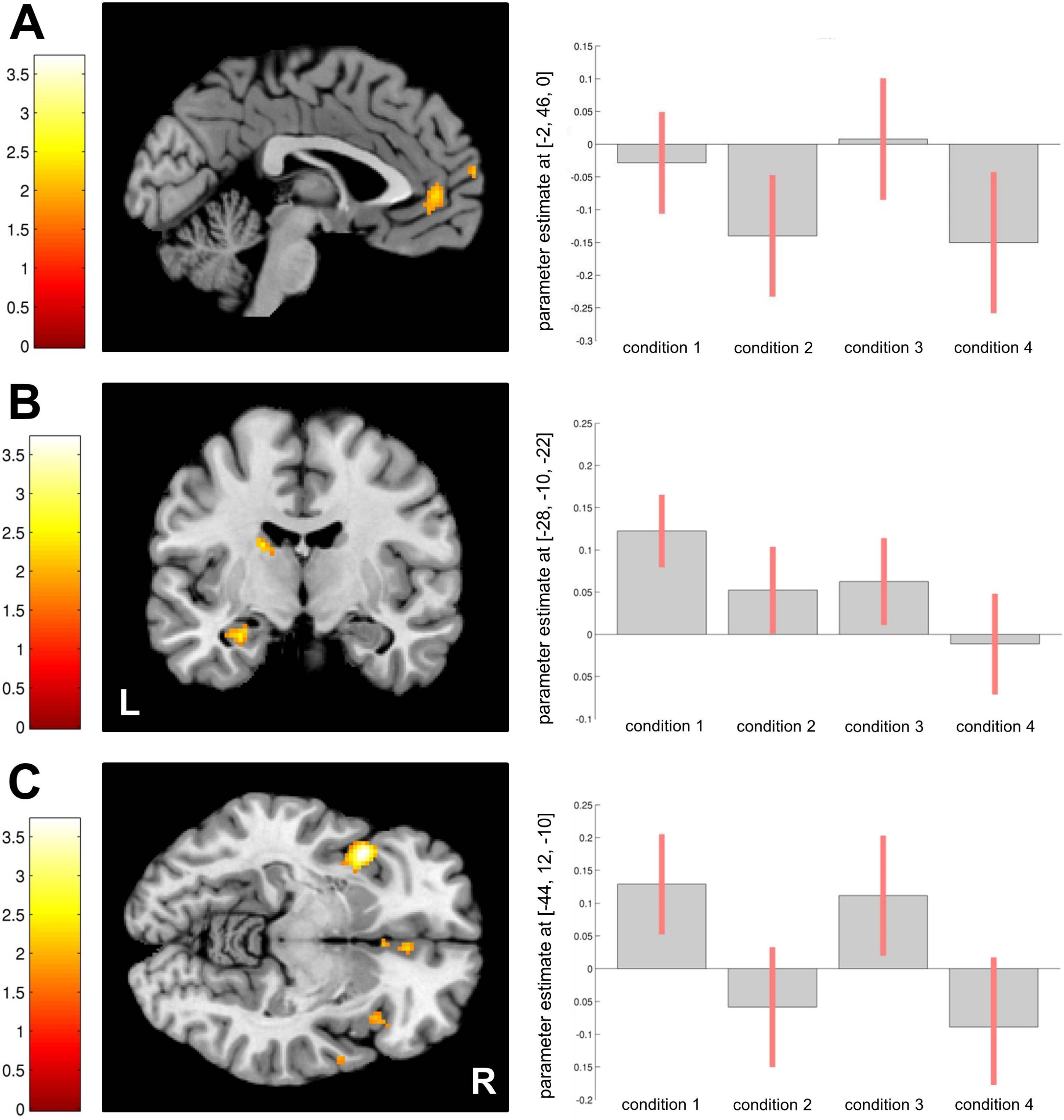

A
B
C
3.5
3
2.5
2
1.5
1
0.5
0
L
R
parameter estimate at [-2, 46, 0]
0.15
0.1
0.05
0
-0.05
-0.1
-0.15
-0.2
-0.25
-0.3
parameter estimate at [-28, -10, -22]
0.25
0.2
0.15
0.1
0.05
0
0.05
-0.1
parameter estimate at [-44, 12, -10]
0.25
0.2
0.15
0.1
0.05
0
0.05
-0.1
0.15
-0.2
condition 1
condition 2
condition 3
condition 4
